# Construction of a Standardized Tongue Image Database for Diagnostic Education: Development of a Tongue Diagnosis e-Learning System

**DOI:** 10.3389/fmedt.2021.760542

**Published:** 2021-12-22

**Authors:** Makoto Segawa, Norio Iizuka, Hiroyuki Ogihara, Koichiro Tanaka, Hajime Nakae, Koichiro Usuku, Yoshihiko Hamamoto

**Affiliations:** ^1^Department of Kampo Medicine, Yamaguchi University Hospital, Ube, Japan; ^2^Yamaguchi Health Examination Center, Yamaguchi, Japan; ^3^Division of Electrical, Electronic and Information Engineering, Graduate School of Sciences and Technology for Innovation, Yamaguchi University, Ube, Japan; ^4^Department of Traditional Medicine, Faculty of Medicine, Toho University, Tokyo, Japan; ^5^Department of Emergency and Critical Care Medicine, Akita University Graduate School of Medicine, Akita, Japan; ^6^Department of Medical Information Science and Administrative Planning, Kumamoto University Hospital, Kumamoto, Japan

**Keywords:** tongue examination, tongue diagnosis, database, e-learning, Kampo, education, malnutrition, glossitis

## Abstract

Tongue examination is an important diagnostic method for judging pathological conditions in Kampo (traditional Japanese medicine), but it is not easy for beginners to learn the diagnostic technique. One reason is that there are few objective diagnostic criteria for tongue examination findings, and the educational method for tongue examination is not standardized in Japan, warranting the need for a tongue image database for e-learning systems that could dramatically improve the efficiency of education. Therefore, we constructed a database comprising tongue images whose findings were determined on the basis of votes given by five Kampo medicine specialists (KMSs) and confirmed the educational usefulness of the database for tongue diagnosis e-learning systems. The study was conducted in the following five steps: development of a tongue imaging collection system, collection of tongue images, evaluation and annotation of tongue images, development of a tongue diagnosis e-learning system, and verification of the educational usefulness of this system. Five KMSs evaluated the tongue images obtained from 125 participants in the following eight aspects: (i) tongue body size, (ii) tongue body color, (iii) tongue body dryness and wetness, (iv) tooth marks on the edge of the tongue, (v) cracks on the surface of the tongue, (vi) thickness of tongue coating, (vii) color of tongue coating, and (viii) dryness and wetness of tongue coating. Medical students (MSs) were given a tongue diagnosis test using an e-learning system after a lecture on tongue diagnosis. The cumulative and individual match rates (%) (individual match rates of 100% (5/5), 80% (4/5), and 60% (3/5) are shown in parentheses, respectively) were as follows: (i) tongue body size: 92.8 (26.4/26.4/40.0); (ii) tongue body color: 83.2 (10.4/20.8/52.0); (iii) tongue body dryness and wetness: 88.8 (13.6/34.4/40.8); (iv) tooth marks on the edge of the tongue: 88.8 (6.4/35.2/47.2); (v) cracks on the surface of the tongue: 96.8 (24.0/35.2/37.6); (vi) thickness of tongue coating: 84.8 (7.2/21.6/56.0); (vii) color of tongue coating: 88.0 (15.2/37.6/35.2); and (viii) dryness and wetness of tongue coating: 74.4 (4.8/19.2/50.4). The test showed that the tongue diagnosis ability of MSs who attended a lecture on tongue diagnosis was almost the same as that of KMSs. We successfully constructed a tongue image database standardized for training specialists on tongue diagnosis and confirmed the educational usefulness of the e-learning system using a database. This database will contribute to the standardization and popularization of Kampo education.

## Introduction

Since tongue findings reflect a variety of systemic disorders, including malnutrition, tongue examination is one of the basic physical examinations. Median rhomboid glossitis, atrophic glossitis, lichen planus, leukoplakia, hairy tongue, and glossodynia are sometimes associated with tongue disorders. Morphological abnormalities, including geographic tongue, fissured tongue, and macroglossia, are also often observed (1–3). These are associated with various pathological conditions, including malnutrition, infectious diseases, systemic diseases, hereditary diseases, age-related changes, and unknown causes (1–3). In particular, atrophic glossitis is associated with malnutrition (4) and is accompanied by a smooth red tongue surface with atrophy of the tongue papilla. Suppression of tongue papilla cell regeneration is caused by insufficient intake or malabsorption of specific nutrients such as iron (Plummer-Vinson syndrome due to iron deficiency anemia) (5), vitamin B12 (pernicious anemia) (6), vitamin B2, and folic acid (7).

Tongue findings are also important in Kampo medicine, which is the traditional Japanese medicine. It is based on traditional Chinese medicine but developed as a unique form in Japan. Kampo has been the backbone of Japanese medicine for more than 1,500 years (8–10). Historically, observation of the tongue as a diagnostic methodology first appeared in China around the Jin (1115–1234) and Yuan Dynasties. The extant first work of tongue diagnosis is the Ao Shi Shang Han Jin Jing Lu (Ao's Records of Golden Mirror on Cold Pathogenic Diseases) written in 1341. It was later brought to Japan and published in 1654. Tongue examination was deeply studied during the Edo period (1603–1868) in Japan (11, 12).

Kampo medicine doctors consider the physical and mental conditions comprehensively through four examinations, i.e., inspection, listening and smelling examination, medical interview, and examination by touch manipulation, to determine the final diagnostic pathology, “sho,” and administer Kampo medication (13, 14). Tongue diagnoses were included in the inspection. Tongue findings are considered to reflect physical and mental conditions. The doctor evaluates the color, morphology, and movement of the tongue body, and the color, morphology, and dryness of the tongue coating, and determines their association with an imbalance of Ki (Qi) (vital life force energy), Ketsu (blood), and Sui (body fluid). Kampo diagnosis is divided into four dichotomic categories:Yin/You (yin/yang), Kyo/Jitsu (deficiency/excess), Kan/Netsu (cold/heat), and Hyou/Ri (exterior/interior) (13–15).

The normal color of the tongue body is defined as light red in Kampo medicine. Compared with normal color, pale, red, deep red, and purple tongues indicate deficiency and cold, heat, advanced heat, and blood stasis, respectively. Normal morphology of the tongue body is defined as a tongue without swelling, atrophy, tooth marks, or cracks. Compared with normal morphology, swelling, atrophy, tooth marks, and cracks indicate water retention and Ki(qi) deficiency, Ki (Qi) deficiency and Ketsu deficiency, Sui retention and/or Ki (Qi) deficiency, and Ketsu deficiency and/or lack of Sui, respectively (16, 17).

In a healthy person, the tongue is kept clean by the action of moistening and cleaning by saliva, chewing movement, mechanical friction of the tongue movement, normal oral flora, and nutritional supply. However, if these functions are impaired, the hygienic environment in the oral cavity deteriorates, and the tongue coating tends to increase. The tongue coating is composed of exfoliated cells, mucus, bacteria, and food residue. A thin white tongue coating is considered normal, while a thick white coating suggests cold, yellow coating suggests heat, and no coating suggests both Ki(Qi) and Ketsu deficiency. Dry tongue coating suggests yang state, while wet tongue coating suggests the yin state in Yin-Yang theory (16, 17).

As mentioned above, tongue diagnosis is a basic examination technique in Kampo medicine; however, it is not easy for beginners to master it. To successfully offer a tongue diagnosis to patients, long-term training in distinguishing various complex tongue findings and understanding pathological conditions is essential. Furthermore, the lack of standard diagnostic criteria hinders accurate tongue diagnosis, which consequently depends on the subjective evaluation of the practitioner. The ambiguity of tongue diagnosis may inhibit the development of Kampo medicine research and the implementation of standardized Kampo medicine education. Therefore, there is a need to develop a modern teaching strategy for the efficient and accurate education of tongue diagnosis techniques. To solve the aforementioned problems, we constructed a database of standard tongue images that could guarantee diagnostic quality and developed a tongue diagnosis electronic learning (e-learning) system. Furthermore, we have proposed an objective method determined by a majority vote to ensure the reliability of the diagnostic information of the tongue images used in the database. The educational advantage of this system is that it can be learned at any place and time repeatedly, and the diagnostic ability of the tongue can be evaluated. The introduction of this learning system is expected to dramatically improve the learning efficiency of tongue diagnosis and promote standardization in tongue diagnosis education.

This research was jointly conducted by KMS and engineering researchers (ERs) who are familiar with educational technology and image recognition. The aims of this study were to construct a database of tongue images, which are annotated with correct labels by KMSs, and to create a tongue diagnosis e-learning system using the database and verify its educational usefulness.

## Materials and Methods

This study was conducted in the following sequence: Step 1: Development of tongue image capture and collection system; Step 2: Collection of tongue image data; Step 3: Evaluation and annotation of tongue image; Step 4: Development of a tongue diagnosis e-learning system; and Step 5: Evaluation of the educational usefulness of this system. This study was evaluated from a scientific and ethical point of view in accordance with the Declaration of Helsinki and the ethical guidelines for medical and health research for humans established by the Ministry of Health, Labor and Welfare of Japan and approved by the ethical review committee at the Clinical Research Center of Yamaguchi University Hospital (Research Approval Number: H27-067-3).

### Step 1: Development of Tongue Imaging Collection System

**Figure 1:**
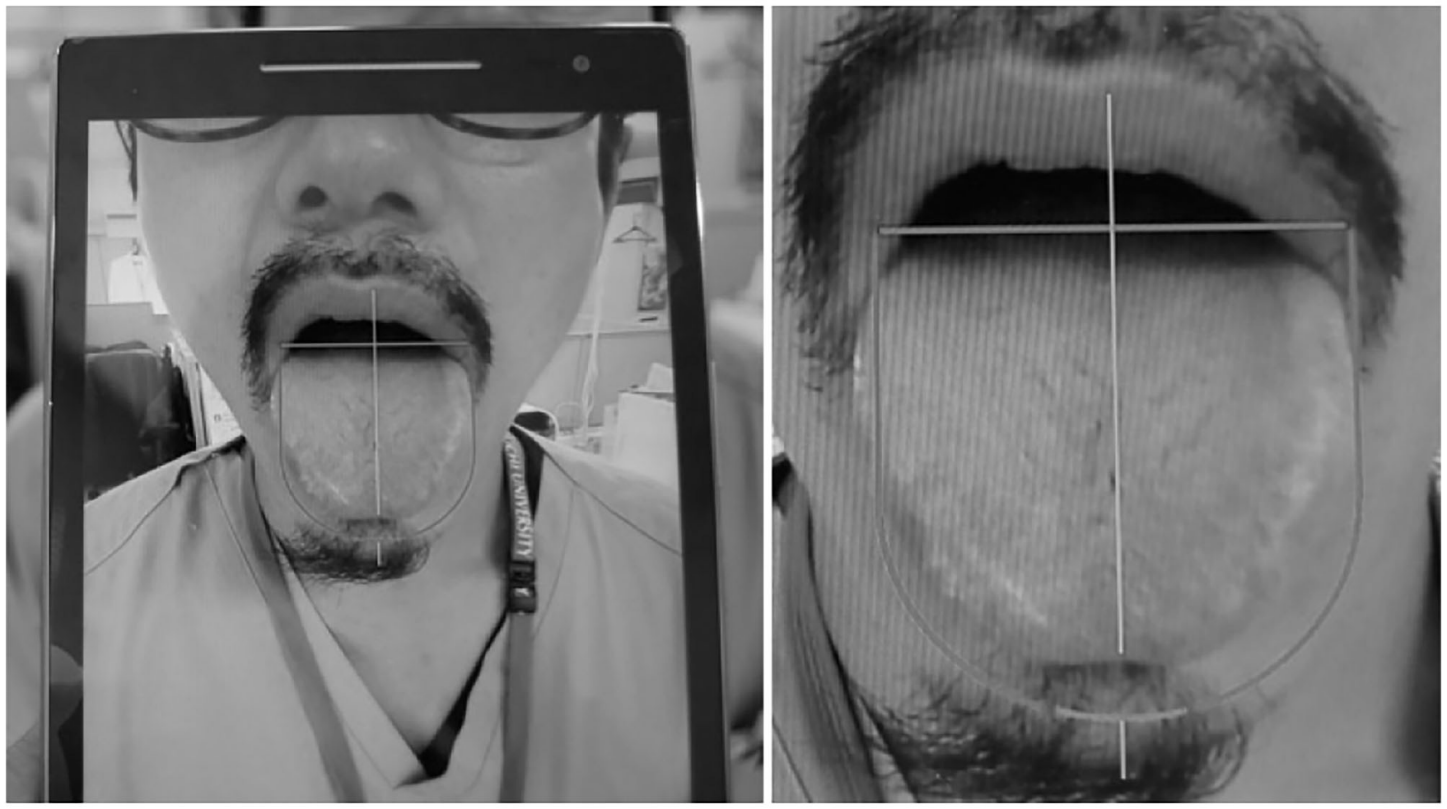
Tablet-type mobile device equipped with a tongue image collection system. Tablet-type mobile device with a camera function that has a “tongue-shaped framework” set on the shooting screen. When shooting the tongue according to the framework, the shooting conditions are naturally unified; thus, the shooting bias due to the difference in the size and direction of the tongue and the manner of tongue presentation can be reduced.

While taking pictures, environmental factors, such as the color tone and illuminance of the lighting, the quality of the shooting device, the shooting method, and the way the subject's tongue sticks out, could affect the image quality. To obtain clear tongue images suitable for interpretation, we developed a tongue image capture and collection system (inventor: Yoshihiko Hamamoto, Norio Iizuka, et al. Japanese Patent Application Laid-Open No. 2019-213652), which can constantly adjust the shooting environment. This system is a tablet-type mobile device with a camera function. The feature of this system is that a tongue-shaped framework is set on the filming screen on the tablet to capture the tongue of the subject. When filming the tongue according to the framework, the filming conditions are naturally unified; thus, biases due to the difference in the size and direction of the tongue and the method of tongue presentation can be reduced. As the filming distance between the subject and the camera is kept constant, defocusing can also be prevented (Figure 1). The tongue images were taken using a tablet-type device (ASUS ZenPad^TM^ 8.0 Z380M, screen resolution: 1,280 × 800 pixels, ASUSTeK Computer Inc.). Regarding lighting, one arm-type desk lamp (MCST-13K, illuminance: 300 lux, fluorescent lamp type: FPL13EX, MAXER DENKI Co., Ltd.) was placed on both diagonal sides of the patient. The color of the fluorescent lamp was “daylight,” which is a whitish light close to natural light. The illuminance in the room where the image was taken was measured with a digital illuminator (TM-205, Tenmars electronics co. ltd.), and we confirmed that the illuminance was maintained at ~800 (745–865) lux.

### Step 2: Collection of Tongue Image Data

Using the tongue imaging collection system, we acquired tongue image data of 125 patients who visited the Kampo Medicine Outpatient Department of Yamaguchi University Hospital (100 patients) and St. Hill Hospital (25 patients). Written informed consent was obtained from all the participants. The inclusion criteria were as follows: (1) patients who visited the Kampo medicine outpatient department of Yamaguchi University Hospital and St. Hill Hospital, (2) patients aged 20 years or older, and (3) patients who obtained written consent voluntarily to participate in the study. Exclusion criteria were patients who did not meet the above inclusion criteria. The data collection period lasted from December 2019 to July 2020. There were 36 men and 89 women in the samples; the patients' ages ranged from 24 to 93 years (60.7 ± 15.0 years).

The photographs were taken in a room with an appropriate illuminance of 700–800 lx, with auxiliary lighting devices attached to both sides of the subject. The image was taken within 10 s of the presentation of the tongue. Shootings were repeated three times independently, and the image data were saved in the computer as anonymized data. A researcher other than the photographer selected the best quality tongue images.

### Step 3: Evaluation and Annotation of Tongue Images

In general, a diagnosis given by only one doctor could be unreliable. If the same diagnosis given by multiple doctors, it indicates high concordance, and the reliability can be regarded as high. In addition, when specialists with vast clinical experience give a diagnosis, its reliability increases. Therefore, in this study, five KMSs with vast clinical experience diagnosed the same tongue images on the same level of display using laptop computers with same specifications at different locations. If the same diagnostic result was given by three or more of the five KMSs, we defined it as the correct diagnosis (the correct answer).

They evaluated eight findings from 125 tongue images: (i) tongue body size, (ii) tongue body color, (iii) tongue body dryness and wetness, (iv) tooth marks on the edge of the tongue, (v) cracks on the surface of the tongue, (vi) thickness of tongue coating, (vii) color of tongue coating, and (viii) dryness and wetness of tongue coating. Individual match rates of 5/4/3 evaluators and cumulative match rates were calculated for each of the eight items. In addition, the diagnostic results for each of the eight items were analyzed.

The diagnosis of the tongue images by five KMSs was performed using the same computer with the same specifications equipped with software that allowed each evaluator to input the diagnostic result. To ensure anonymity and objectivity, the diagnostic data were analyzed by the ERs.

All the five KMSs were proficient in Kampo practice and certified by the Japan Society for Oriental Medicine. The average age of the KMSs (48–62 years old) was 55 ± 5.5 years old [mean ± standard deviation (SD)], and each one belonged to a different medical institution.

Since the tongue diagnosis results were confirmed by majority vote of the KMSs, the results were high-quality information, ensuring reliability and objectivity. A tongue image with a high diagnostic match rate was considered a high-quality image showing the typical characteristics of each item and suitable for educational use. In this way, a tongue image database with a high-quality, correct labels was constructed.

### Step 4: Development of Tongue Diagnosis e-Learning System

**Figure 2:**
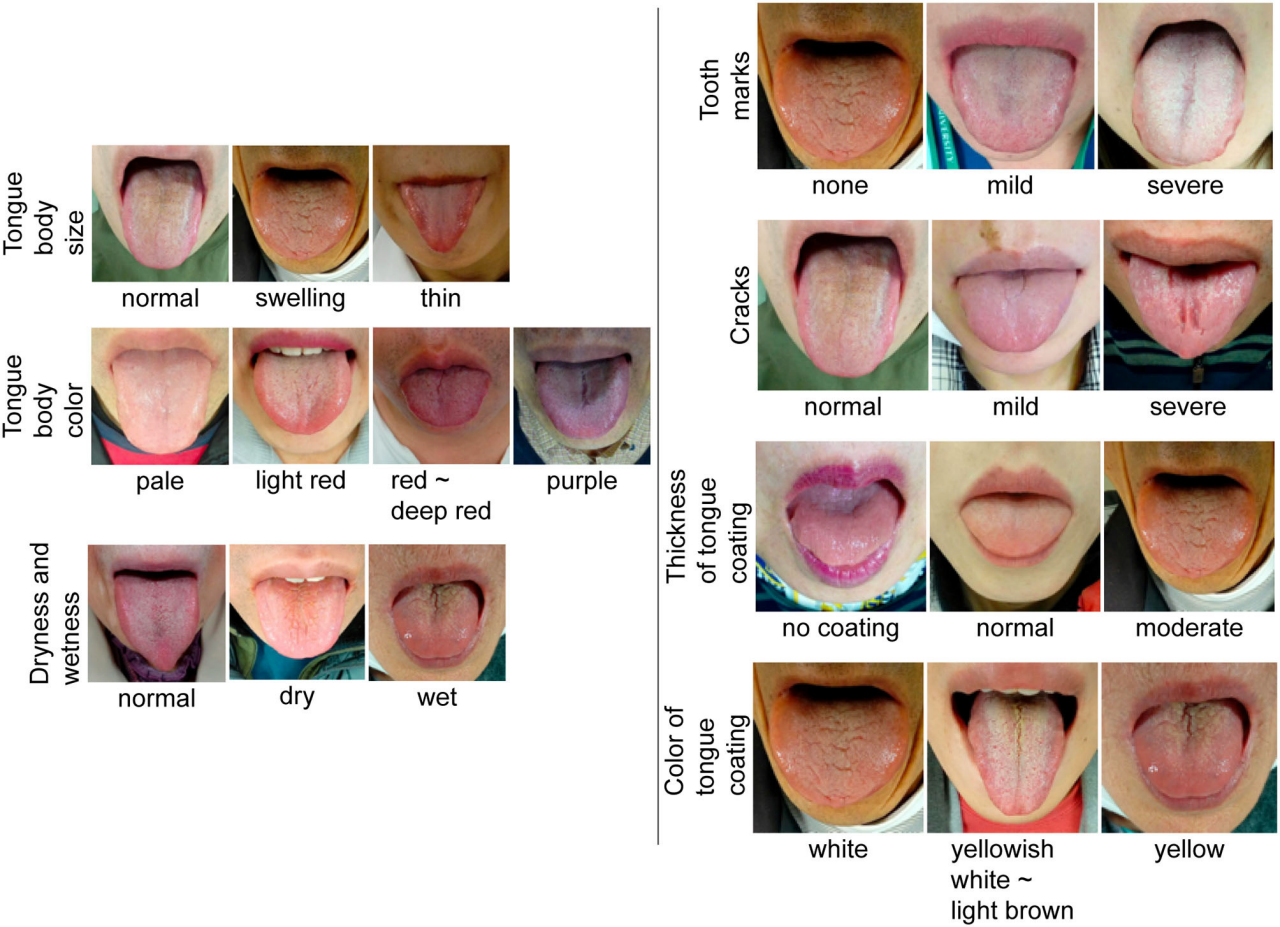
Standard tongue image. Standard tongue images for each item are presented. As the images of dryness and wetness of the tongue body overlap with those of the tongue coating, they have been combined into one.

**Figure 3:**
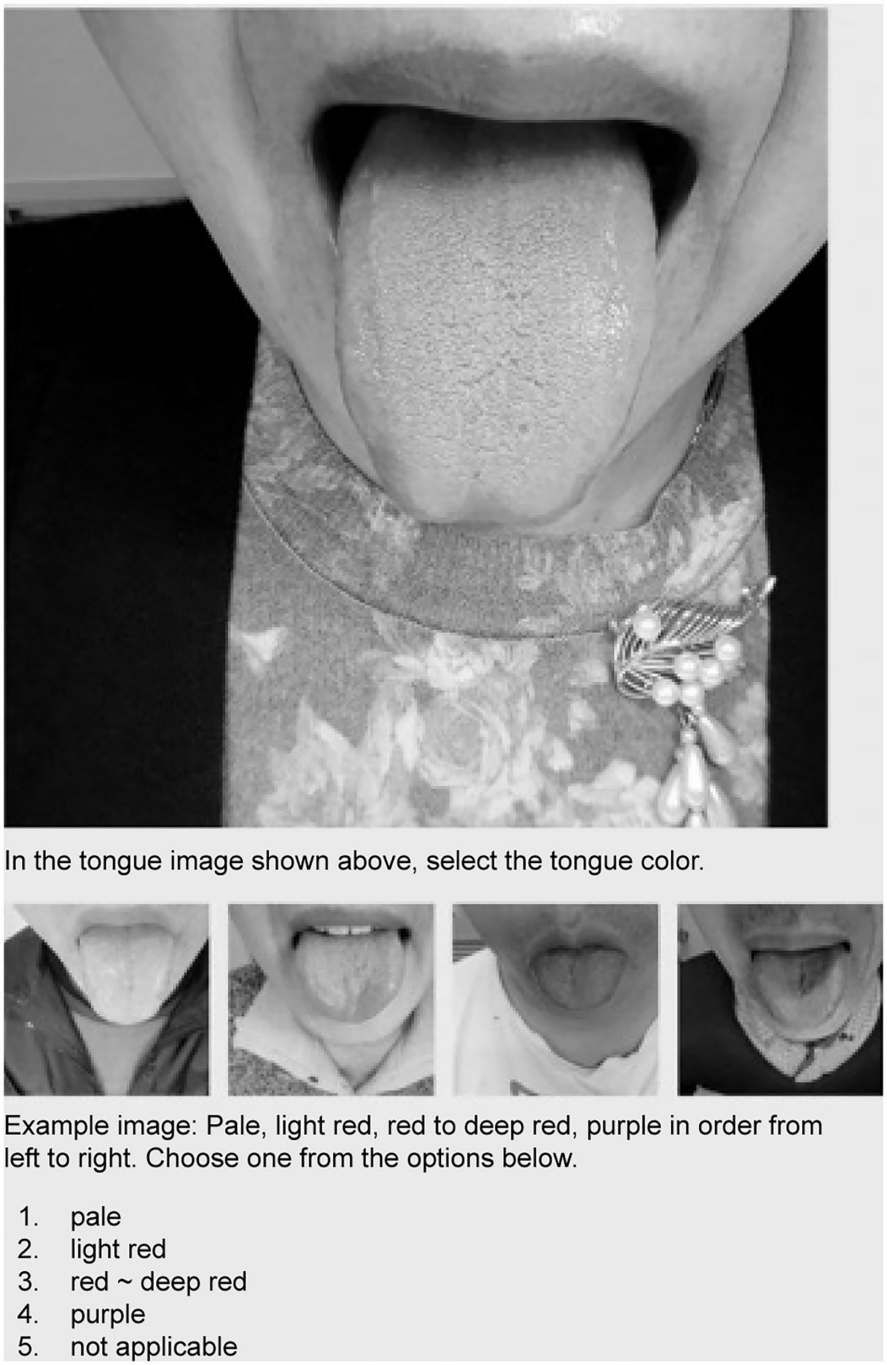
Screen of tongue diagnosis test using e-learning system. Candidates select an answer by referring to the sample image.

**Figure 4:**
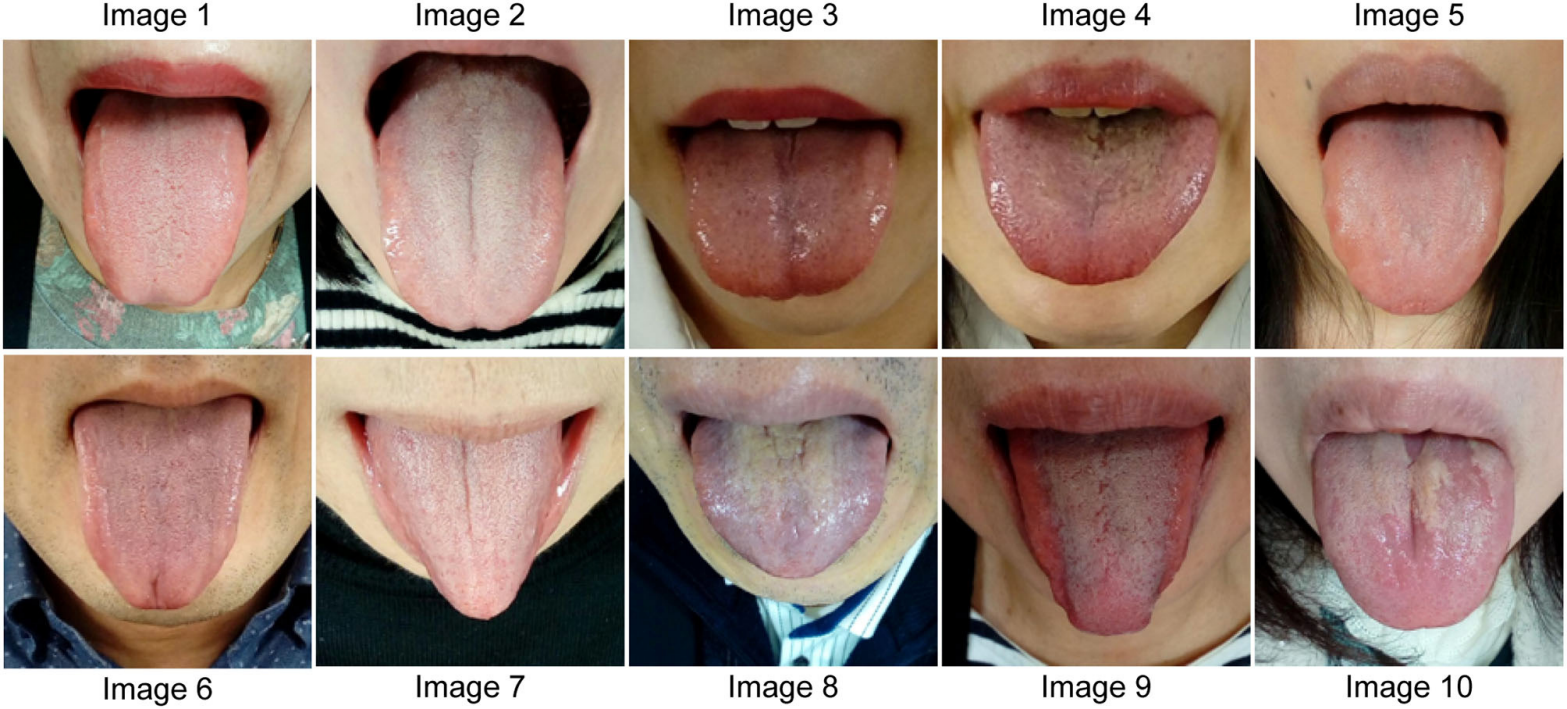
Tongue images for the tongue diagnosis test. Tongue images of 10 people used in the tongue diagnosis test are shown. The features of each tongue image are listed in Table 1.

We developed a tongue diagnosis e-learning system that utilizes images from a quality-guaranteed tongue image database. This e-learning system makes it possible to not only learn without restrictions on time, place, and equipment but also perform a self-evaluation of tongue diagnostic ability. The procedure is as follows: (1) access an e-learning website; (2) pre-learning: confirm the eight standard tongue images displayed on their device before taking the test (Figure 2); (3) tongue diagnosis test: questions about the presented tongue image are shown in Figure 3; (4) send answer data and check grades: immediately after sending the answer data, check the score, correct/incorrect display of all questions, and 8-items radar chart; (5) confirmation of deviation value in the test group: When taking the test in a group, the average value, maximum score, minimum score, own score, frequency distribution, and radar chart of the group are displayed. This allows the learners to see their grades in a group.

### Step 5: Verification of the Educational Usefulness of Tongue Diagnosis e-Learning System

To evaluate the educational usefulness of this learning system, we created a test of 80 questions (eight findings in the tongue images obtained from 10 participants) (Table 1; Figure 4), which was administered to 112 MSs of Yamaguchi University School of Medicine after they received a lecture on tongue diagnosis. The five KMSs took the same test without a lecture. The MSs took the test with a smartphone. From the student's answer data, the average score, score distribution, and correct answer rate for each of the eight items were calculated, and the degree of acquisition of tongue diagnosis ability was evaluated. The answers of the MSs were compared with those of the five KMSs.

**Table 1:** Correct answer of the 80 questions used for the tongue diagnosis test.

	**Image 1**	**Image 2**	**Image 3**	**Image 4**	**Image 5**	**Image 6**	**Image 7**	**Image 8**	**Image 9**	**Image 10**
Tongue body size	Normal	Normal	Normal	Swelling	Swelling	Normal	Thin	Normal	Normal	Swelling
Tongue body color	Light red	Pale	Red–dark red	Purple	Light red	Light red	Light red	Purple	Red–dark red	Light red
Tongue body dryness and wetness	Normal	Normal	Wet	Normal	Normal	Normal	Normal	Dry	Normal	Normal
Tooth marks	None	None	None	None	Mild	None	None	None	None	None
Cracks	None	None	Mild	Mild	None	None	None	Mild	Mild	None
Thickness of tongue coating	Normal	Normal	Normal	Normal	Normal	Normal	Normal	Moderate	Moderate	Moderate or higher with some peeling
Color of tongue coating	White	White	White	Yellowish white–light brown	White	White	White	Yellowish white–light brown	White	Yellowish white–light brown
Dryness and wetness of tongue coating	Normal	Normal	Wet	Normal	Normal	Normal	Normal	Wet	Normal	Wet

*The correct answer was automatically determined by a majority vote of 5 KMSs. KMS, Kampo medicine specialists*.

### Statistical Analysis

Values are expressed as the mean ± SD. The significance of differences was determined using the Student's *t*-test. (PASW^®^ Statistics 18). Statistical significance was set at *P* ≤ 0.05.

### Description of Medical Terms in Kampo Medicine

#### The Three Substance Categories: Ki, Ketsu, and Sui

Ki (Qi) is the vital force energy that is the source of movement of the human body, which moves blood, water, and organs, and regulates the mental state, autonomic nervous system, endocrine system, and immune system. Ketsu (blood) refers to the red fluid in the body and is considered a vital nutrient substance, which nourishes bodily tissues and organs and ensures adequate mental function. Sui (body fluids) refers to the clear fluids in the body, which moisten and nourish the body and includes saliva, gastric juices, joint fluids, tears, mucus, sweat, and urine. A state in which Ki, Ketsu, and Sui circulate in a well-balanced manner is considered healthy, and if any of them is excessive, insufficient, or stagnant, illness will occur (13–15).

#### Disease Condition Categories: Yin-You, Kyo-Jitsu, Kan-Netsu, and Hyou-Ri

The four dichotomic categories are used to classify the patient's disease condition or state and determine the Sho, which means symptoms, signs, or evidence. If the repairing responses shown by the patient against his/her disease condition are feverish, active, and excitatory, the patient is said to be in You-Sho (positive or active pattern). Conversely, if the responses shown by the patient are chilly, inactive, and inhibitory, the patient is said to be in Yin-Sho (negative or passive pattern). Further, if the repairing responses shown by the patient against his/her disease condition are strong or fully active, the patient is said to be in Jitsu-Sho (excess pattern), while if they are weak or hollow, he/she is said to be in Kyo-Sho (deficiency pattern). If the repairing responses shown by the patient against his/her disease condition are febrile, the patient is said to be in Netsu-Sho (heat pattern), while if they are chilly, the patient is said to be in Kan-Sho (cold pattern). If the part where the reaction against the disease appears is near the body surface, it is called Hyou-Sho (exterior pattern), but if it is deep inside the body, it is called Ri-Sho (interior pattern) (13, 14).

## Result

### Match Rate of Tongue Diagnosis Results of Five KMSs

The match rate of the tongue diagnosis results of the five KMSs for 125 tongue images was calculated for each item (Table 2). The cumulative match rate (%) (cumulative match rate of three or more evaluators) and individual diagnosis match rate (%) (match rates of 5/4/3 evaluators are shown in parentheses) for each of the eight items are as follows: (i) tongue body size: 92.8 (26.4/26.4/40.0); (ii) tongue body color: 83.2 (10.4/20.8/52.0); (iii) tongue body dryness and wetness: 88.9 (13.6/34.4/40.8); (iv) tooth marks on the edge of the tongue: 88.8 (6.4/35.2/47.2); (v) cracks on the surface of the tongue: 96.8 (24.0/35.2/37.6); vi) thickness of tongue coating: 84.8 (7.2/21.6/56.0); (vii) color of tongue coating: 88.0 (15.2/37.6/35.2); and (viii) dryness and wetness of tongue coating: 74.4 (4.8/19.2/50.4). The mean values (± standard deviation) of match rates of 5/4/3 evaluators were 13.5 ± 8.1%, 28.8 ± 7.6%, and 44.9 ± 7.5%, respectively.

**Table 2:** Diagnostic match rates of five KMSs.

	**Individual match rates (%)**			**Cumulative match rates (%)**
	**100% (5/5)**	**80%** **(4/5)**	**60%** **(3/5)**	**60% or more**
Tongue body size	26.4	26.4	40.0	92.8
Tongue body color	10.4	20.8	52.0	83.2
Dryness and wetness of tongue body	13.6	34.4	40.8	88.8
Tooth marks on the edge of the tongue	6.4	35.2	47.2	88.8
Cracks on the surface of the tongue	24.0	35.2	37.6	96.8
Thickness of tongue coating	7.2	21.6	56.0	84.8
Color of tongue coating	15.2	37.6	35.2	88.0
Dryness and wetness of tongue coating	4.8	19.2	50.4	74.4
Mean value ± SD	13.5 ± 8.1	28.8 ± 7.6	44.9 ± 7.5	87.2 ± 6.7

*The match rate of the tongue diagnosis results of the five KMSs with respect to the tongue images of 125 patients was calculated for each of the eight items. The individual match rate shows the match rate of five out of five KMSs, four out of five KMSs, and three out of five KMSs. The cumulative match rate shows the sum of the individual match rates of three out of five KMSs. KMS, Kampo medicine specialists; SD, standard deviation*.

The cumulative diagnostic concordance rate for three or more of the five patients was highest for cracks on the surface of the tongue at 96.8%, lowest for dryness and wetness of tongue coating at 74.4%, and the average value for all the items was 87.2 ± 6.7%. The cumulative diagnostic concordance rate for four or more of the five patients was the highest for cracks on the surface of the tongue at 59.2%, the lowest for dryness and wetness of tongue coating at 24.0%, and the average value for all the items was 42.3 ± 15.7%.

### Analysis of the Diagnosis Results for Each of the Eight Items by Five KMSs

Images with matching tongue diagnosis results for three or more of the five KMSs were analyzed for each of the eight items in the 125 tongue images (Table 3). (i) Size of the tongue: 56.9% for normal, 37.1% for swelling, and 6% for thin; (ii) color of the tongue: 18.3% for pale red, 52.9% for light red, 13.5% for red to deep red, and 15.4% for purple; (iii) dryness and wetness of the tongue body: 58.6% for normal, 17.1% for dryness, and 24.3% for wetness; (iv) tooth marks: 66.7% for none, 26.1% for mild, and 7.2% for severe; (v) cracks: 45.5% for normal, 44.6% for mild, and 9.9% for severe; (vi) thickness of the tongue coating: 1.9% for none, 49.1% for normal, 44.3% for moderate, 3.8% for moderate or higher with some peeling, and 0.9% for thick; (vii) color of the tongue coating: 58.2% for white, 35.5% for yellow to light brown, 5.5% for yellow, and 0% for dark brown to black; (viii) dryness of the tongue coating: 44.1% for normal, 23.7% for dryness, and 32.3% for wetness. In this study, about half of the patients exhibited normal color and morphology, and the other half showed pathological findings.

**Table 3:** Diagnosis result of tongue image determined by majority vote of three or more out of five KMSs.

	**Diagnosis result**	**Number of patients**	**Ratio (%)**
Tongue body size	Thin	7	6.0
	Normal	66	56.9
	Swelling	43	37.1
	Total	116	100
Tongue body color	Pale	19	18.3
	Light red	55	52.9
	Red or deep red	14	13.5
	Purple	16	15.4
	Total	104	100
Tongue body dryness and wetness	Normal	65	58.6
	Dry	19	17.1
	Wet	27	24.3
	Total	111	100
Tooth marks on the edge of the tongue	None	74	66.7
	Mild	29	26.1
	Severe	8	7.2
	Total	111	100
Cracks on the surface of the tongue	Normal	55	45.5
	Mild	54	44.6
	Severe	12	9.9
	Total	121	100
Thickness of tongue coating	None	2	1.9
	Normal	52	49.1
	Moderate	47	44.3
	Moderate or higher with peeling	4	3.8
	Thick	1	0.9
	Total	106	100
Color of tongue coating	White	64	58.2
	Yellowish white to brown	39	35.5
	Yellow	6	5.5
	Dark brown to black	0	0
	Not applicable	1	0.9
	Total	110	100
Dryness and wetness of tongue coating	Normal	41	44.1
	Dry	22	23.7
	Wet	30	32.3
	Total	93	100

*The diagnosis results of the tongue image determined by a majority vote of three or more out of five KMSs were analyzed for each of the eight items. Tongue images that were not decided by majority vote were excluded. KMS, Kampo medicine specialists*.

### Evaluation of Diagnostic Consistency of Five KMSs

To evaluate the consistency of diagnoses of the five KMSs, the responses to the 80-question test after database construction were compared with those reported at the time of database construction, and the consistency rate was calculated. The average match rates for the eight items were as follows: (i) tongue coating size: 74.0 ± 16.7%; (ii) tongue coating color: 58.0 ± 19.2%; (iii) tongue coating dryness and wetness: 68.0 ± 20.5%; (iv) tooth marks on the edge of the tongue: 70.0 ± 29.2%; (v) cracks on the surface of the tongue: 64.0 ± 15.2%; (vi) thickness of tongue coating: 58.0 ± 19.2%; (vii) color of tongue coating: 46.0 ± 20.7%; and (viii) dryness and wetness of tongue coating: 72.0 ± 17.9%. The consistency on the color of the tongue body and color of the tongue coating tended to be low. The average consistency rate for all items was 63.8 ± 10.1%.

### Evaluation of Learning Effect Using e-Learning System

#### Tongue Diagnosis Test Score

**Figure 5:**
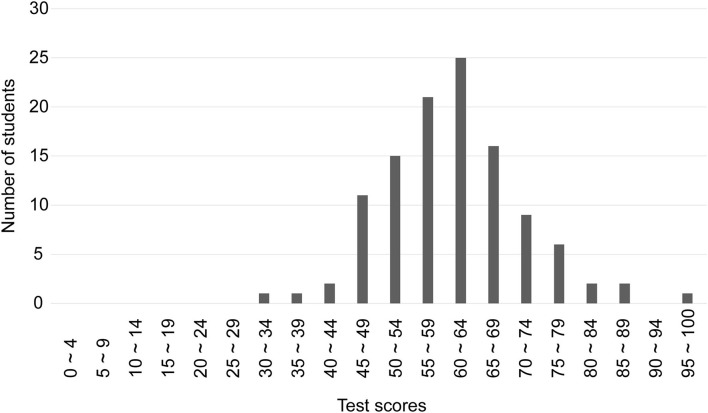
Distribution of test scores for 112 medical students. The scores of the tongue diagnosis test of 112 MSs showed a normal distribution from the lowest score of 34 points to the highest score of 100 points. The average score was 60.9 ± 10.7 points.

The scores of the tongue diagnosis test of 112 MSs showed a normal distribution from the lowest score of 34 points to the highest score of 100 points (Figure 5). The average score was 60.9 ± 10.7 points. In contrast, the average score of the five KMSs was 57.5 ± 12.8. There was no statistically significant difference between the two scores (*p* = 0.239). Assuming that a score of 60 points or more was used as the criterion for acquiring tongue diagnostic ability, it was considered that 46.4% of the MSs were able to acquire it.

#### Comparison of Diagnostic Match Rates Between MDs and KMSs

Table 4 shows the average diagnostic match rate for 10 tongue images for each of the eight MS and KMS items. The diagnostic match rate of the MSs was similar to that of KMSs, and no statistically significant difference was observed in any of the eight items. The diagnostic match rates (%) of MSs and KMSs in the eight items are as follows: (i) tongue body size: 62.9 ± 15.2, 68.0 ± 23.4 (*p* = 0.57), (ii) tongue body color: 65.1 ± 32.6%, 60.0 ± 28.2 (*p* = 0.71), (iii) tongue body dryness and wetness: 65.3 ± 16.1, 66.0 ± 23.2 (*p* = 0.94), (iv) tooth marks on the edge of the tongue: 68.9 ± 19.6, 60.0 ± 28.3 (*p* = 0.43), (v) cracks on the surface of the tongue: 53.0 ± 22.9, 64.0 ± 32.4 (*p* = 0.39), (vi) thickness of the tongue coating: 59.4 ± 20.7, 51.0 ± 25.1 (*p* = 0.43), (vii) color of tongue coating: 59.1 ± 24.6, 42.0 ± 25.7 (*p* = 0.15), and (viii) dryness and wetness of tongue coating: 54.5 ± 19.3, 50.0 ± 27.1 (*p* = 0.57).

**Table 4:** Comparison of mean diagnostic match rate for 10 tongue images between 112 MSs and five KMSs.

	**Tongue body size**	**Tongue body color**	**Dryness and wetness of tongue body**	**Tooth marks on the edge of the tongue**	**Cracks on the surface of the tongue**	**Thickness of tongue coating**	**Color of tongue coating**	**Dryness and wetness of tongue coating**
Medical students (*n* = 112)	62.9 ± 15.2	65.1 ± 32.6	65.3 ± 16.1	68.9 ± 19.6	53.0 ± 22.9	59.4 ± 20.7	59.1 ± 24.6	54.5 ± 19.3
Kampo specialists (*n*= 5)	68.0 ± 23.4	60.0 ± 28.2	66.0 ± 23.2	60.0 ± 28.3	64.0 ± 32.4	51.0 ± 25.1	42.0 ± 25.7	50.0 ± 27.1
*p*-value	0.57	0.71	0.94	0.43	0.39	0.43	0.15	0.67

*The mean diagnostic match rate for the 10 tongue images of MS and KMS is shown. The diagnostic match rate of MS was similar to that of KMSs. There were no statistically significant differences in any of the eight items. KMS, Kampo medicine specialists; MSs, medical students*.

## Discussion

Tongue diagnosis in Kampo involves accurately interpreting complex and diverse tongue findings and appropriately identifying Kampo medical pathology indicated by those findings. Although tongue diagnosis is an important technique in Kampo medicine, it has the following problems: (i) there are few diagnostic criteria, (ii) it depends on a subjective evaluation, and (iii) the diagnosis result varies depending on the diagnostic criteria (18, 19). This makes it difficult to measure the tongue morphology and color of the patients. In addition, different criteria are used by individual medical doctors who determine the final diagnosis on the basis of tongue findings. These results are inconsistent in terms of diagnosis among medical doctors. We speculate that some of the reasons for the variability in diagnostic results are due to the lack of standardization of diagnostic and training methods in tongue diagnosis.

Recently, standardization of diagnostic methods for tongue examination in Kampo has been attempted (20, 21). Standardized educational programs for tongue diagnosis are also important in modern Kampo medical education. Currently, basic education in Kampo medicine is provided in all medical universities in Japan (22). However, little research has been conducted on educational methods for tongue diagnosis, and only a few research studies have been conducted in China (23, 24). The traditional learning method of tongue diagnosis is to learn by observing the patient's tongue in daily clinical practice, but it takes a long time and effort to acquire this ability. Therefore, there is a need to develop a modern teaching strategy for the efficient and accurate education of tongue diagnosis techniques. To solve the above problems, we developed a standardized database of tongue images and proposed the utilization of a tongue diagnosis e-learning system.

This new system is underlined by the following concepts: (i) Simplification, visualization, and iterative learning of knowledge necessary for learning tongue diagnosis skills and (ii) management of learning level using an e-learning system. The first concept is to train the image interpretation ability by dividing it into eight fields. By training for the reading and interpretation ability of individual components, it becomes possible to make integrated judgments quickly without much effort. Using this method, the learner can intuitively grasp the judgment criteria of the color and morphology, which are difficult to evaluate quantitatively, and it can improve the discriminating ability. Second, this is a learning management system that uses e-learning. E-learning enables two-way communication via the Internet. The advantages are as follows: (i) a large number of learners can study repeatedly at any time and place; (ii) the quality of education is uniform and not influenced by the quality of the instructor; (iii) inclusion of rare cases; (iv) able to centrally manage the progress of learning and feedback and objectively evaluate the degree of learning. These features are not found in conventional learning methods and are extremely useful not only because the learning content can be standardized and streamlined but also the diagnostic ability, which is the learning goal, can be evaluated.

In recent years, many studies on tongue diagnosis, such as tongue diagnostic equipment (25–27), automatic diagnostic system using artificial intelligence (28–30), and remote diagnosis using smartphones (31), have been conducted. The diagnostic ability by computers is rapidly improving, and the feasibility of standardization of tongue diagnosis using machines has been sought (32). However, little research has been conducted on educational methods and equipment to improve the ability and skill of human tongue diagnosis. Recently, the usefulness of flipped classroom using an e-learning program in Kampo education has been reported (33). In addition, it should be noted that we previously conducted a WEB-based test for medical students to evaluate the learning effect of Kampo medicine in collaboration with several medical universities and reported its usefulness in learning management using information and communication technology (34, 35).

In this study, the percentages of images that matched for 5/4/3 KMSs were 13.5, 28.8, and 44.9%, respectively. These results suggest that there are some differences in the diagnostic ability of the KMSs. It is presumed that in daily clinical practice, there are a few tongues with classical findings, as shown in a textbook. Additionally, this result might be due, in part, to an individual's ability to discriminate and understand tongue colors. In this study, the average match rate for tongue diagnosis of eight items for the five KMSs was unexpectedly low [63.8 ± 10.1% (SD)] between the first and second tests, and it tended to be the lowest for an item related to color among the eight items. There are individual differences in the perception of colors, and the frequency of color blindness is present in 8% of men and 0.4% of women (36). Based on previous findings (36) and our present findings, we suggest that the ability to diagnose color is unstable. In addition, the ability to discriminate colors, as well as visual acuity, declines with age. In this study, the color discrimination ability and visual acuity of the evaluators were not examined, but the evaluators who exhibited extreme deterioration of visual acuity were not included. Oji et al. evaluated the color discrimination ability of 68 Kampo medical practitioners using the Farnsworth-Munsell 100 Hue test tongue color images and found that tongue color diagnosis significantly differed between subjects with <10 years of experience and >10 years of experience, and practitioners with >10 years of experience could maintain a consistent diagnosis of tongue color regardless of their age (37).

To the best of our knowledge, this is the first study to use e-learning for tongue examination education. To verify the educational usefulness of this system, 112 MSs who took a lesson on tongue diagnosis were administered a tongue diagnosis test. We found that the scores of the MSs were almost similar to those of the KMSs, indicating the high performance of our constructed e-learning test in evaluating the learning effect in tongue examination. In addition, we found that by taking a lesson on tongue diagnosis, it is possible for MSs to temporarily acquire diagnostic knowledge that is almost the same as that of a KMS. However, further studies may be necessary to verify whether this high ability for tongue diagnosis can be maintained for a long period of time.

## Conclusion

We constructed a high-quality tongue image database and developed a tongue diagnosis e-learning system. Since this system can repeatedly self-learn at any time and place, it is possible to acquire efficient tongue diagnosis techniques. This learning management system using information and communication technology is expected to contribute to the modernization and standardization of tongue examination education.

## Data Availability Statement

The raw data supporting the conclusions of this article will be made available by the authors, without undue reservation.

## Ethics Statement

The studies involving human participants were reviewed and approved by the Ethical Review Committee at the Clinical Research Center of Yamaguchi University Hospital. The patients/participants provided their written informed consent to participate in this study. Written informed consent was obtained from the individual(s) for the publication of any potentially identifiable images or data included in this article.

## Author Contributions

MS and NI collected tongue image data. MS, HO, and YH performed data analysis. MS, NI, HO, and YH contributed to conception and writing of the manuscript. KT, HN, and KU critically revised the manuscript. All authors contributed to the construction of database and e-learning system and approved the final manuscript.

## Funding

This research was carried out using a research grant from the Japan Kampo Medicine Education Foundation in 2020.

## Conflict of Interest

The authors declare that the research was conducted in the absence of any commercial or financial relationships that could be construed as a potential conflict of interest.

## Publisher's Note

All claims expressed in this article are solely those of the authors and do not necessarily represent those of their affiliated organizations, or those of the publisher, the editors and the reviewers. Any product that may be evaluated in this article, or claim that may be made by its manufacturer, is not guaranteed or endorsed by the publisher.

## Abbreviations

ERs: engineering researchers
KMS: Kampo medicine specialists
MSs: medical students
SD: standard deviation.
